# Master’s degree in sexual and reproductive health: enhancing career development opportunities for midwives in Mali

**DOI:** 10.1186/s12909-023-04853-6

**Published:** 2023-11-21

**Authors:** Cheick S. Sidibé, Tanya Brückner, Prisca Zwanikken, Anke van der Kwaak, Lalla Fatouma Traoré, Ousmane Touré, Jacqueline E.W. Broerse, Marjolein Dieleman

**Affiliations:** 1 National Training Institute in Health Sciences, Bamako, Mali; 2 Athena Institute for Research on Innovation and Communication in Health and Life Sciences, Vrije Universiteit, Amsterdam, Netherlands; 3 Royal Tropical Institut, Amsterdam, Netherlands; 4 Department of Education and Research in Public Health, Faculty of Medicine and Odontostomatology, Bamako, Mali

**Keywords:** Midwives, Education, Master, Career, Degree, Continuous professional development, Preservice, Opportunities, Mali

## Abstract

**Background:**

Midwives’ contribution to improving outcomes for women and newborns depends on factors such as quality of pre-service training, access to continuing professional development, and the presence of an enabling work environment. The absence of opportunities for career development increases the likelihood that health professionals, including midwives, will consider leaving the profession due to a lack of incentives to sustain and increase motivation to remain in the field. It also limits the opportunities to better contribute to policy, training, and research. This study aimed to assess the influence of a Master in Sexual and Reproductive Health (SRH) at the INFSS on midwives’ career progression in Mali.

**Methods:**

This mixed methods study was conducted using an online questionnaire, semi-structured interviews, and a document review. The study participants included graduates from two cohorts (*N* = 22) as well as employers, managers, and teachers of the graduates (*N* = 20). Data were analysed according to research questions, comparing, and contrasting answers between different groups of respondents.

**Results:**

The study revealed that graduates enrolled in the programme primarily to improve their knowledge and skills in management and public health. The graduates’ expected roles are those of programme and health project manager and participation in planning and monitoring activities at national or sub-national level. The managers expected the programme to reflect the needs of the health system and equip midwives with skills in management and planning. The Master enhanced opportunities for graduates to advance their career in fields they are not usually working in such as management, research, and supervision. However, the recognition of the master’s degree and of the graduates’ profile is not yet fully effective.

**Conclusion:**

The master’s degree in SRH is a capacity building programme. Graduates developed skills and acquired advanced knowledge in research and management, as well as a postgraduate degree. However, the master programme needs to be better aligned with health system needs to increase the recognition of graduates’ skills and have a more positive impact on graduates’ careers.

## Background

Despite the progress accomplished over the last decades, maternal mortality remains a major public health concern in low-and-middle-income countries (LMICs), particularly in sub-Saharan Africa, which accounts for approximately 66% of maternal deaths worldwide [1]. In Mali, the maternal mortality ratio (MMR) was estimated at 325 deaths per 100,000 live births in 2018 [2]. This ratio is well above the United Nations’ Sustainable Development Goal (SDG) 3, which aims to reduce the global maternal mortality ratio to less than 70 per 100,000 live births by 2030 [3]. Midwifery care is an effective solution to decreasing maternal and newborn mortality in LMICs [4, 5]. Midwives’ ability to contribute to improving outcomes for women and newborns depends on factors such as quality of pre-service training, access to continuing professional development, and the presence of an enabling work environment [6, 7].

In addition, their contribution to policy, training and research is important but needs strengthening [6]. Overall progress towards universal health coverage (UHC) and the SDGs is weakened without midwives’ participation in advising health policies due to the exclusion of vital contextual and experiential knowledge related to sexual and reproductive health (SRH) generally and maternal and child healthcare specifically [8]. Moreover, despite 80% of low-income countries reporting to have midwife leaders at some level, ranging from positions at the national Ministry of Health to positions at health facilities [6], they often lack preparation and adequate training [6, 9]. Although promising developments have been made in midwifery education recently, such as improved quality of training due to International Confederation of Midwives’ core competencies guidelines for midwifery training [10], professional career development opportunities for higher strategic positions in LMICs are still limited [6, 11]. The limited opportunities for career development increase the likelihood that health professionals, including midwives, will consider leaving the profession due to a lack of incentives to sustain and increase motivation to remain in the field, and limit the opportunities to better contribute to policy, training, and research [6]. Therefore, there is a global call to strengthen midwifery training and extend it beyond the clinical domain into training to develop competencies in leadership, management, and research to provide career opportunities in decision-making, training and research in SRH, and in particular in maternal health [12].

In this context, the World Health Organisation (WHO) Global Strategic Directions for Nursing and Midwifery 2021–2025 [13], informed by the State of the World’s Midwifery 2021 report [6], identified four intervention areas to optimise midwives’ contribution to reducing maternal and child mortality, improving SRH and other population health goals: education, jobs, leadership, and service delivery. Each area has a strategic direction: 1) educating enough midwives with competencies to meet population health needs; 2) creating jobs, recruiting, managing, and retaining midwives where they are most needed; 3) strengthening midwifery leadership throughout health and academic systems; and 4) ensuring midwives are supported, motivated, and equipped to contribute to their service delivery settings safely and optimally. The strategic directions include policy priorities based on evidence and reflect effective health-workforce strengthening approaches using a health labour-market lens. The Global Strategic Directions for Nursing and Midwifery was endorsed through a resolution at the 74^th^ World Health Assembly in 2021 [14]. The policy priorities aim to guide member states, including Mali, in their actions to fully enable the contribution of midwives to meet population health needs.

Efforts in Mali to improve midwives’ quality of training and thus contribute to strengthening the health system has been marked by two significant changes: 1) a change of entry level requirements into midwifery training, made conditional on the baccalaureate diploma, which came into force in 1997; and 2) linking midwifery training to the Licence (bachelor) – Master – Doctorate (LMD) scheme, which has been in force since 2012. The training lasts three years and confers a bachelor’s degree in midwifery. There is no master’s level training in midwifery and the possibility of access to doctoral training is extremely limited. As a result, midwives are rarely involved in activities such as management, research, and postgraduate midwifery training. Although midwives who become trainers receive some initial training prior to teaching, there is no academic requirement for them to hold such a position in Mali. Thus, most midwives who are in training positions do not have a master’s degree and are only involved in undergraduate training.

Despite the global call to increase midwives’ access to managerial positions and high-level teaching and researcher posts, this is confronted with a double limitation in Mali: firstly, the small number of places offered in management, research, and training in master’s programmes and employment structures in general, and secondly, the limited number of midwives with a master’s or doctorate degree. For example, there is no position at the Ministry of Health for midwifery practice management. In 2018, only four midwives were reported to be working at a national service level, without any specification on the position they held. None of them were reported to be working in the Ministry of Health [15]. There is no indication that this situation has changed significantly.

In order to improve midwives’ career opportunities, in 2017 a two-year, master’s degree in Sexual and Reproductive Health for midwives and nurses was offered and later approved by the Ministry of Higher Education. This study aimed to assess the influence of the master’s degree programme in SRH on graduates’ career progression in Mali. Career progression is defined as roles in leadership, management, research, and training positions, or advanced clinical practice positions. The research questions were: What profile, role and employment opportunities did the master’s degree in SRH graduates, and employers expect from the master’s degree programme? What skills did they learn from the master’s degree programme in relation to their current employment? How did the master in SRH influence the graduates’ current employment situation?

## Methods

A convergent mixed methods study was conducted using an online cross-sectional survey, semi-structured interviews, and a document review. Data were collected in October 2019 and February 2021 by two trained data collectors from the university of Bamako with master’s degree in public health and SRH, and by the first author.

### Setting of the study

The management tasks usually performed by midwives in Mali are of health care units within health structures (although specific data are missing). They are most often promoted to these positions based on their clinical expertise or seniority. Consequently, they are often ill-prepared for these responsibilities and have no formal training opportunities to qualify for such positions. While the University of Bamako offers a Research Master in SRH, and the programme is open to all medical professionals, its fees are high. Consequently, acceptance into the programme is viewed as more competitive for midwives, thus acting as a barrier for them to enrol into the Master. The University’s master’s in public health (MPH) programme admits 20 to 25 students per cohort, divided between different tracks in the second year of the programme, including the SRH track. The most recent cohort in the SRH track consisted of three doctors and one nurse. To date, no midwives have ever been admitted to the programme.

Since 2006, the national training institute in health sciences (INFSS), the only public paramedical training institute in Mali, has offered a MPH, pedagogy, and medical specialties to midwives, contributing to a solution to midwives’ limited access to higher education and training. Upon a pressing request from the Association and Council of Midwives, the INFSS opted to offer a master’s level vocational training programme in SRH. Between 2015- 2017, INFSS developed the master programme, which was offered to students from 2017 onwards. In comparison to the Master offered at the University of Bamako, the fees are more affordable ($640 at the INFSS annually versus $4,000 annually at the University) and acceptance is limited to nurses and midwives, making it a more attractive option for these cadres. Nurses are included as they often share midwifery tasks in Mali. The West African Health Organisation (WAHO) curriculum was used to develop the programme and the use of the WAHO-curriculum also facilitates accreditation to WAHO sub-regional standards. An opinion widely shared amongst managers at the INFSS, midwifery organisations and midwives themselves, is that the Master in SRH is an opportunity for midwives to access a master’s degree without embarking on other medical specialties such as ENT (ear, nose, and throat), ophthalmology, and so on. However, training in areas such as public health, health service management, pedagogy or surgical assistance are already existing opportunities at master’s degree level open to midwives that can contribute to strengthening their role in management, research, or clinical practice. Therefore, the question that arose was the influence of training in SRH on midwives’ career progression.

A situation analysis was conducted by the INFSS in 2015 to discuss the option to develop a SRH-master programme, followed by a workshop at the INFSS on its findings, to contextualise the WAHO’s curriculum and define expected graduate competencies. This workshop brought together a broad range of stakeholders, including stakeholders from the Human Resources Department of the Ministry of Health, the midwifery council and association, and the Ministry of Higher Education, as well as teachers, programme managers from INFSS, and gynaecologists and practising midwives. The workshop participants debated over the type of competencies that needed to be prioritised in the curriculum, and participants had to decide between a public health profile and a clinical profile for the SRH master’s degree. The public health profile refers to the development of skills that are considered important to leadership and management roles, such as general skills in management, planning, monitoring and evaluation, and an introduction to research in SRH and programme development for SRH issues at community level. This profile would broaden employment perspectives beyond care provision and include posts such as SRH programme manager, SRH researcher, SRH planner, consultants, and senior management roles in the health system. The clinical content aims to provide midwives with a deeper clinical competence than a bachelor's degree level. The clinical profile consists of skills in management of obstetric emergencies, post-abortion care, management of pregnancy-related pathologies, and skills such as instrumental deliveries and use of ultrasound equipment.

The workshop participants considered both profiles important to contribute to improved sexual and reproductive health generally and maternal and child health specifically. Therefore, a two year, four-semester curriculum that combined both profiles was developed and later approved by the Ministry of Higher Education. It is intended for midwives with at least three years of work experience and nurses with midwifery experience, who would be full-time students through face-to-face learning modalities. In the 2019 and 2020 cohorts, 33 of 36 students in total graduated with a master’s degree in SRH.

### Study population and sampling

The study participants included graduates from the 2018 and 2019 cohorts (cohort 1 and 2, respectively) of the Master in SRH, as well as employers, managers/colleagues, and teachers of those graduates. For the survey, all graduates from the graduate list were invited to participate (*N* = 32). An invitation letter providing the details and purpose of the study was sent to the INFSS to request a contact list of graduates from the two cohorts. All contacts were verified by a cell phone text message and all graduated who agreed to participate were included in the study. For the semi-structured interviews (SSI), graduates were purposely selected based on their profile (nurse or midwife), occupation before they started the master (employed, not employed, involvement clinical or public health activities) and cohorts (cohort 1 and 2). We included employers and managers/colleagues in the SSI to gain insight in their views on the master’s degree and the graduates. The employers and manager/colleagues were selected from different levels of the health system and from the public and private sectors, based on the list of employment structures obtained from the graduates. Teachers of the Master’s in SRH were selected according to their profile: clinical and public health, and the SRH training programme managers at the INFSS were included in the study.

### Data collection

For the *online questionnaire* a link was sent to all graduates of the programme who were willing to participate in the study (*N* = 22) by text message or email in February 2021; an accompanying cover letter outlined the details of the study and assured the participants of anonymity. To maximise the response rate, researchers made telephone calls and sent follow-up emails. A reminder message and call were made every week for four weeks after the initial message. The questionnaire consisted of 48 topics grouped into demographic characteristics, occupational status, acquisition of knowledge and skills during the master’s, professional advancement after the course, and perception of the quality, relevance, and attractiveness of the course.

In addition, individual *semi-structured interviews* (SSI) were conducted. Data collection was done by telephone (*N* = 11) or face-to-face (*N* = 15) when feasible, using semi-structured interview guides. Questions were asked about the improvement in knowledge and skills resulting from the master’s programme, the implementation at work of the skills acquired during the programme, perceptions of professional advancement attributable to the programme, and perceptions of the quality and relevance of the programme. Interviews were recorded with participants’ consent.

The online questionnaire and SSI guide were adapted from questionnaire developed by Zwanikken et al. [16] and piloted prior to data collection in January 2021 with a sample of graduates of the master’s in reproductive health at the University of Bamako, resulting in minimal changes made in wording and format. Specific interview guides were developed for employers, managers, and teachers, which were piloted and adapted according to feedback prior to data collection.

The *document review* aimed to explore and understand the course’s contents and duration, programme objectives, organisation of teaching, assessments, internships, and course materials. Documents reviewed (*N* = 30) included the situational analysis report, the INFSS’s and WAHO’s training programs, the INFSS’s internal regulations document, course manuals and course materials, training reports, students’ internship reports and end-of-study dissertations.

### Data analysis

Descriptive analysis of the data was done using IBM SPSS Statistics version 27. SSIs were conducted in French and tape recorded. They were transcribed and then analysed using NVivo qualitative software version 12 (QSR International. Nvivo 12. Burlington 2018). The first author inductively developed codes after reading a few transcripts. These codes were discussed with and validated by a second researcher. The codes were then applied to all transcripts and additional emerging codes were considered. The data were analysed according to research questions, comparing, and contrasting answers between different groups of respondents. For document reviews, data was extracted using a data extraction checklist, which was developed based on the research questions.

### Findings

The main results are presented below, according to the following themes: participants’ characteristics; the SRH curriculum content; graduates’ expected profile, role, and employment; graduate skills; and actual role and employment. Each group of key-informants are presented as one, as no difference within the groups were noticed.

### Participants’ characteristics

#### Survey participants

A total of 14 students enrolled in the programme in 2017, and 21 in 2018. Three students did not graduate and were not included in the study. Of the 32 graduates, 22 participants completed the survey, and 10 did not respond to the invitation despite the follow up, resulting in a response rate of 68.75%. Table 1 shows the participants’ socio-demographic and professional characteristics. One of the respondents was male, the others female. The median age of graduates at the end of training was 37 years, with a minimum age of 29 and maximum of 48. All had clinical working experience with 7 years median clinical working experience prior to the training. Seventeen out of 22 respondents had professional educational backgrounds in midwifery, four in nursing.

**Table 1:** Characteristics of the survey participants

Characteristics	N
**Sex**	
Male	1
Female	21
**Age at the time of survey, in years**	
Median	37
Range	19
Minimum	29
Maximum	48
**Clinical working experiences in SRH**	
Mean	6.6
Median	7
Minimum	0
Maximum	13
**Previous qualification**	
Midwife	17
Nurse	4
Other	1

#### Interview participants

A total of six graduates and 20 non-graduate key informants participated in the SSI (see Table 2). Graduates had professional educational backgrounds in midwifery (5/6) and nursing (1/6); five were women. The median age of participants at the end of training was 34 years (min = 31 and max = 45).

**Table 2:** Participants in the qualitative study

Overview of different groups of key informants	N
Graduates	6
Employers - (Health) Facility managers - NGO (working in SRH) managers - (Health) programme managers	4
Colleagues and line managers: - Midwives (colleague and line manager) - Doctors (gynaecologists, general practitioners) - Public health workers (with master’s in public health)	6
Training managers: - Deputy director in charge of pedagogy at INFSS - Head of the SRH department at INFSS - Coordinator of the Master in SHR	3
Teachers	4
Midwifery Council and Midwifery Professional Association	2
Policy makers MOH, department of HRH	1
Total	26

### Expectations regarding the profile, role and employment of graduates

Participants were asked about their expectations about the graduate profile (clinical versus public health), role, and employment of SRH Master graduates. Based on the findings, it became clear that the vision and expectations regarding the master’s programme varied highly among respondents.

The survey and the SSI revealed that most graduates and key informants were not familiar with the master and had no clear idea of the intended profile for graduates prior to the training. According to students and school managers participating in SSI, there was no specific information provided to students prior to enrolment. The document review has shown that there were no brochures or information on the websites apart from an announcement on the availability of the training. In the survey, 19/22 of graduates expected a training programme for a public health profile, including planning and/or management skills and competencies, as well as communication skills. In comparison, the clinical profile appeared less desirable (*N* = 3). Most SSI participants, including employers, teachers, and students, thought that the public health profile would allow midwives to broaden their knowledge, thinking, and scope of work beyond clinical practice. This would enable them to contribute more or differently to the reduction of maternal mortality:

“*The midwives only do clinical practice and generate data. For example, at the health centre we provide care and assist women giving birth. But for the woman to come to the ANC (antenatal care clinic), we [often] need to work within communities. For this, you need skills that a midwife does not have.”* [Midwife, master degree graduate]

Another reason why participants preferred a public health profile to a clinical profile is due to trained clinicians’ anticipated hesitancy to relocate from urban to rural areas, thus not realistically increasing the scope of care:

“*The clinical profile at specialisation (master’s degree) can always strengthen the service delivery. But the problem is not the availability of [clinically competent] midwives, but [rather] their distribution. If midwives are trained with more skills, like gynaecologists, there will be conflict of competences. “Simple” midwives already don’t want to go to remote areas, [so] it’s not [anticipated that] the midwives with more clinical training will agree to go there. They will all want to stay in urban areas and in hospitals where there are already enough midwives and gynaecologists.*” [Midwife, master degree graduate]

Nevertheless, a few teachers and students (*N* = 3) thought that a clinical profile was desirable for capacity building, broadening the scope of work to include more technical skills, for example, as well as greater autonomy for midwives. They also thought it would be beneficial to remain closer to midwifery practice, in order to prevent loss of midwives to other specialty positions such as in public health.

Currently, from participants in SSI, there are uncertainties around the possible clinical employment of graduates. Graduates do not expect to return to clinical posts and the midwifery associations and council representatives prefer midwives trained at master level not to work at the clinical level. They believe it should lead to improved involvement of graduates in SRH policy making and taking up other decision-making positions, in which midwives have had scant opportunity to participate so far. According to the majority of the participants, most decision-making posts are currently for medical doctors:

“*With the master’s degree, I can participate more in decision making. For example, for the recent health reform, there were no midwives when it was being drafted. There were only directors, deputy directors, people from the ministry, and all doctors. These decision makers can forget things or not understand the factual issues in maternal health*.” [Midwife, master degree graduate]

Table 3 shows graduates’ expected type of employment activities from the survey.

**Table 3:** Types of activities (graduate preferences) from the survey

Tasks/activities	N
Management of SRH programmes and projects	18
Research	10
Teaching	7
Advanced clinical practice	7
Other	1

From the survey, most graduate participants (*N* = 19/21) preferred the public health profile to the clinical profile and their ideal jobs was managers of SRH programmes and projects, researchers and teachers. In relation to these jobs, the most preferred place of employment would be in referral health centres (CSREF), regional and general directorates of health at the Ministry of Health, and at NGOs working in SRH. Most graduate participating in the SSI were also in favour of public health profile. They expect to perform tasks, such as the design, planning, monitoring, evaluation, and supervision of SRH interventions. According to health facility managers, these positions are currently filled by people who have not received specialist SRH training at a master’s level:

“*The people who occupy these positions [at the moment] are often midwives, general practitioners, and nurses. There are no specialists. This is to tell you that we make do with what we have, as we don’t have the ideal people. The graduates should be inserted at these levels, and we must let them play their role.*” [Employer, CSREF, specialist in public health]

### SRH curriculum contents

The document review shows that the programme includes compulsory modules and ends with a dissertation. The modules were divided over four semesters. Table 4 shows the competences, course modules, and their duration (WAHO – Harmonized training curriculum for nurses and midwives in ECOWAS region. West African Health Organization; 2014). In total 280 h (14%) are spend on the clinical profile and 1376 h (69.4%) on the public health and management profile. The internship represents 330 h (16.6%) based on public health competencies and management.

**Table 4:** Competencies, modules, and duration of courses contents

Competencies	Modules	Duration (hours)
***Semesters 1 and 2***		
Ensure the management and monitoring of health programs and projects	Management and quality of care	140
	National Health System	100
	Management of Health Programs and Projects	60
	Monitoring and Evaluation of Health Programs and Projects	80
Promote the health of individuals, families, and communities	Gender and health law	120
	Community Health	180
	Socio anthropology	40
	Implementation of promotional care	60
Conduct research in the health	Biostatistics and epidemiology	100
	Communication, IT and English	220
	Health research	30
	Scientific writing	30
Internship: Health promotion/community health		90
***Semesters 3 and 4***		
Analyse the function of graduates in SRH	RH / FP policy and strategies	40
Promote the sexual and reproductive health of the individual, the couple, the family and the community	Sexual and reproductive health of adolescents and young people	40
Provide care for common gynaecological and obstetric conditions	Obstetric gynaecological pathologies	40
	Obstetric and neonatal emergencies care	40
	Management of newborn pathologies	40
	Reproductive system dysfunction	160
Manage reproductive health services, programmes and projects	Engineering and management of a RH / FP service	100
Develop research in sexual and reproductive health	Research methodology in sexual and reproductive health	80
Internship: management of gynaecological and obstetric conditions		240
Writing a dissertation		3 months

### Acquired and applied skills

In the SSI, graduates indicated that the skills they developed the most are those related to conducting research in SRH; identifying reproductive health needs, planning, and monitoring community health actions, accordingly; conducting reproductive health programmes and projects; and analysing and integrating legal and gender aspects into reproductive health activities. The participants in the survey also indicated skills development in research, management, and monitoring. The Table 5 outlines the graduates’ opinions about the acquisition of knowledge and skills during the master’s and the implementation of these skills in their present jobs. Regarding clinical skills, more than half of the graduates reported to not have learned or further developed their skills during the Master. This is corroborated in interviews with the graduates as illustrated in this excerpt:

*“As far as clinical education is concerned, we haven’t learned anything new. We were taught the same things that we already mastered from the bachelor level. For example, newborn resuscitation...”* [Midwife, Master degree graduate]

**Table 5:** Graduates’ opinions on acquisition of knowledge and skills and implementation in the job

Acquisition of knowledge and skills during the course (%)	I have not developed this skill in the Master^a^	I have somewhat developed this skill due to the master	I have developed these skills due to the master to a large extent
Identify the reproductive health needs of the individual, couple, family, and community	0	4	18
Plan, implement, monitor, and evaluate SRH interventions	4	6	12
Analyse gender and legal aspects of reproductive health	2	5	16
Diagnose common gynaecological and obstetric conditions, obstetric and neonatal emergencies	8	7	7
Treat common gynaecological and obstetric conditions	12	5	5
Provide emergency obstetric and neonatal care	12	5	5
Prevent common gynaecological and obstetric conditions	5	6	11
Manage a reproductive health care unit	5	3	14
Plan the activities of a reproductive health service	3	3	16
Conduct a reproductive health programme/project	1	7	13
Monitor and evaluate reproductive health projects/programmes	2	8	13
Conducting studies in SRH	3	5	14
Academic writing	3	9	9
Use of skills during the job (%)	I don’t use it/it isn’t part of my job	I sometimes use this skill	I often use this skill
Identify the reproductive health needs of the individual, couple, family, and community	3	2	17
Plan, implement, monitor, and evaluate SRH interventions	6	3	13
Analyse gender and legal aspects of reproductive health	5	5	12
Diagnose common gynaecological and obstetric conditions, obstetric and neonatal emergencies	5	12	5
Treat common gynaecological and obstetric conditions	7	11	4
Provide emergency obstetric and neonatal care	7	8	7
Prevent common gynaecological and obstetric conditions	5	10	7
Manage a reproductive health care unit	11	4	7
Plan the activities of a reproductive health service	6	3	13
Conduct a reproductive health programme/project	5	3	14
Monitor and evaluate reproductive health projects/programmes	5	3	14
Conducting studies in SRH	3	5	14
Academic writing	8	5	8

^a^I did not learn it or I learnt it from previous training/experience, not due to the master

The most used skills from the survey were those related to planning and monitoring (cf Table 5). However, most graduates in the SSI reported having few opportunities to implement their newly acquired skills because of the jobs and positions they held after graduation. Nonetheless, in SSI, they all reported increased self-confidence and more critical reflection on their practices as well as an improved attitude towards patients as a result of the master’s degree.

### Outcomes post-graduation and application of skills acquired during training

At the time of the study, 19 out of 22 graduates were working as full-time salaried employees. The Master in SRH has led to a change in the employment and role of graduates, as demonstrated in Table 6. Additionally, the programme has led to an increase in salary for more than half of the graduates who participated in the survey (Table 7).

**Table 6:** Survey participants’ employment and roles after completing the master in SRH

Characteristics	N
**Occupational context**	
Hospital/CSREF	9
NGO, not clinical	4
General Directorate of Health (DGS)/ regional Directorate of Health (DRS)	4
Schools	2
Unemployed	2
Research Institutions	1
Total	22
**Activities/tasks (Note: tasks can be multiple)**	
SRH promotion (not clinical)	11
Clinical care	8
Education / Training	6
Supervisor	5
Health information system management	5
SRH programme management	5
Research in SRH	3
Other	1

**Table 7:** Changes in graduates’ occupation and tasks

Professional advancement after the course	N
Leadership	18
Change on activities/tasks	10
Change in salary	12

Before the programme, the graduate employment level was mainly in first level health structures such as the community health centres. These are the first contact service for most users, especially in rural areas. Graduates also worked in referral health centres with health care tasks like deliveries, antenatal care, and family planning services. Three graduates were unemployed. After the programme, most graduates (*N* = 15) worked in central or regional services such as at regional health directorates or referral health centres, and midwife training institutes.

**Fig. 1:**
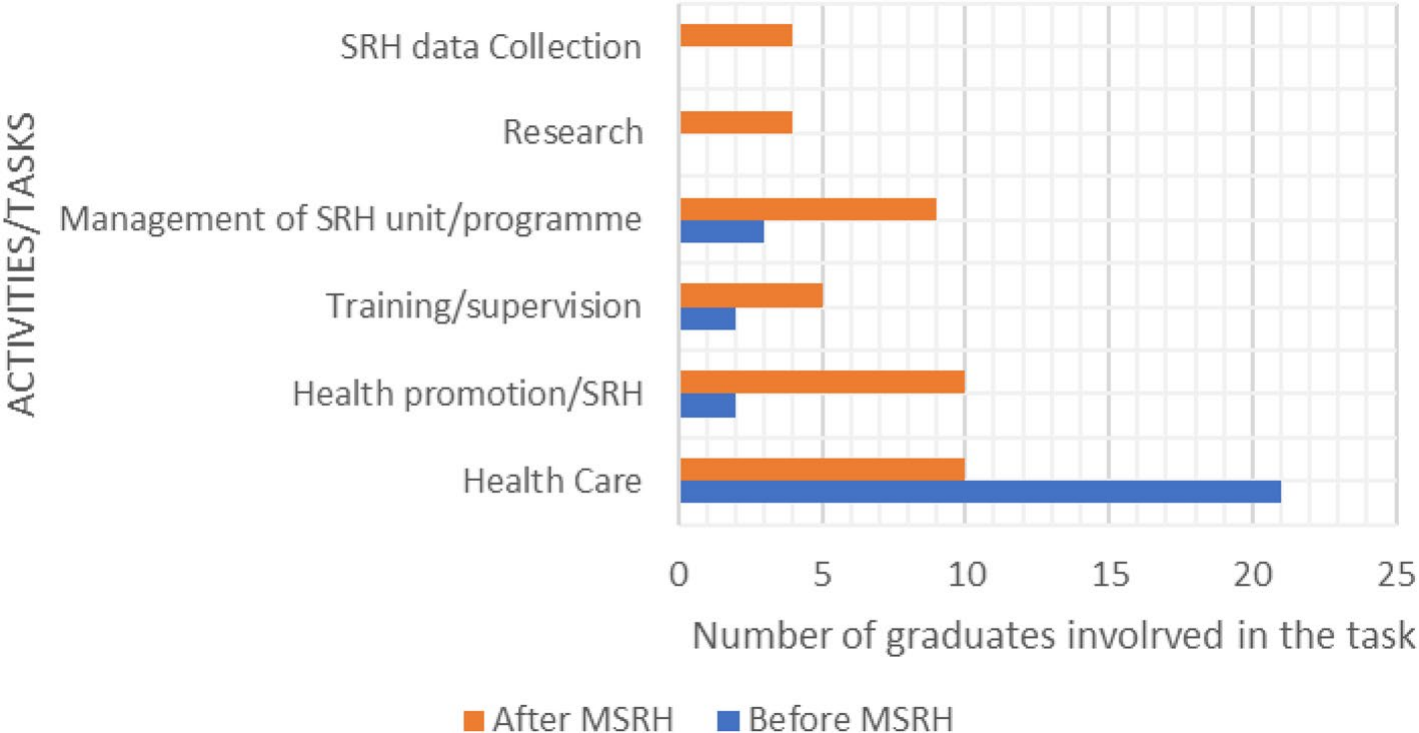
Pre-master’s and post-master’s tasks/activities

Most graduates were working in public health-related jobs or in training. The new tasks and functions were more oriented towards the promotion of SRH activities, supervision, training, and teaching. Their involvement in management and research activities remained low. There was also a decrease in clinical activities (see Fig. 1).

During SSI, several graduates reported having struggled to be accepted by employers because of an unclearly defined graduates’ profile and the lack of posts open for midwives with master’s in SRH. A graduate explains her interaction with employer in public service in the following excerpt:

“*When I arrived, the manager asked me what we were supposed to do, what we could be used for. I made a job description and a detailed description of the skills developed during the Master for him to understand*.” [Midwife, master degree graduate]

From the employers’ perspectives, difficulties relate to lack of knowledge of the graduate employment profile. They think the employers needed to be more involved in the design and be better informed about the training.

“*I think that to integrate them, [the training institution] should discuss it with the health officials, the Regional Health Directors who could well advocate for and use the competencies of the trainees. There should be alignment between what they learned at school and what they are called upon to do at the district level, at the directorate level. This is essential. We need to know more about what they have learned.”* [Employer, DRS]

Most participants considered the Master in SRH appropriate for current employment in public health and service and programme management. However, the content is not adequate for advanced clinical care.

## Discussion

The study aimed to assess the relevance and influence of the Master in SRH on midwives’ career progression. The findings show that the master in SRH provides midwives with an opportunity to develop skills beyond clinical practices and bachelor level. The demand for higher education for midwives has increased, especially in LMICs, where higher educational opportunities are often limited [17]. The master in SRH at INFSS provides midwives with opportunity to develop skills beyond the bachelor level and clinical practice. The clinical and public health purpose of the master was meant to suit the context, as there is not yet an agreed upon role by all stakeholders. But the lack of agreement on the profile hampers clarity for employers and for graduates.

The Master in SRH has influenced graduates’ careers positively, with careers being taken beyond the conventional concept of upward hierarchical movement. The Master enhanced opportunities for graduates in four main ways. First, it enhanced opportunities by boosting the graduates’ confidence and aspirations and this translated into what graduates describe as increased self-confidence. This allows graduates to aspire, apply and compete for positions that were previously considered inaccessible and reserved for other professional categories. Second, the respondent graduates reported development of a range of skills not available at bachelor level. Although these were self-reported, the feeling of increased skills is arguably an indication that the master permitted development in areas that were unavailable to midwives before the master in SRH and prepared them to take up positions previously reserved for higher trained professional. For example, although the importance of including midwives in leadership positions is noted repeatedly, several barriers to accessing these positions are reported [6, 18]. The lack of availability of such positions, the challenging relationships with colleagues and gender-based barriers to access are amongst prevalent global issue [19]. However, to increase midwives’ access to these positions, they first need to be trained accordingly [6, 13, 18, 20]. This opportunity is provided through the master in SSR. Third, the master in SRH has influenced graduate midwives’ employment and career paths, allowing them to move towards management-, training- and research-related tasks. Midwives being trained in these skills will lead to graduate midwives leaving clinical care. However, they are more likely to remain in SRH field in Mali albeit at a higher level. Lastly, the training led to a salary increase for graduates working in the civil sector. This is arguably a signal that the post-graduate level training is valued. However, further training in any profession would result in an increase in salary in the civil sector. Consequently, due to the difficulties in receiving recognition for the skills learnt from the Master, midwives seeking a salary increase could be pushed to pursue further education in other professions that lead them away from midwifery. Professional recognition of skills earned from further training is vital in ensuring commitment to one’s profession and one’s motivation to pursue higher qualifications while remaining in one’s own technical field of expertise (in this case in SHR in Mali). Receiving such recognition contributes to both personal and professional development within one’s field [21, 22].

The recognition of the master’s degree and the graduates is not yet fully effective. This is reflected in the difficulty experienced by some graduates in finding placement after graduation. However, this difficulty seems to be more related to insufficient communication about the program prior to its launch, rather than to a real opposition to the training and the graduates. Yet, employers’ involvement in and opinions about labour market needs and subsequent programme development are crucial to job creation for graduates. It should also be noted that it was the midwives’ council, rather than the employers and the institutions, that heavily influenced the opening of the master. This made it more difficult to match training with employers’ needs and graduates’ employment opportunities.

To achieve its objectives, the Master in SRH needed communication and discussion with potential employers as well as a more defined implementation strategy to ensure that the role of midwives with a master’s degree in SRH is integrated into the health system. This has been possible in other LMICs for successful implementation of the master’s degree for nurses and midwives, such as in Eswatini and the Caribbean [23, 24]. Factors that enabled those successes included meetings with policy, practice, and community experts to define the role and how it fit into population health priorities and goals. Meetings with these stakeholders also included planning implementation strategies and scope of practice [23]. Such considerations and practices could potentially also have benefitted graduates in our study. It would have also benefitted the graduates to lobby for clear SRH specialist positions in order to increase the availability of jobs with these skills requirements.

### Study strengths and limitations

The study intended to report on the broad influence of master in SRH on graduates’ career progression. All the assessments are self-assessments. However, using information from multiple sources including from peers and employers, documents, and self-reported answers from the graduates strengthens our findings (triangulation). The relatively low number of graduates and the time since graduation may be a limit to our study. However, it gives a good indication of the outcomes of the training. Including employers that have employed SRH graduates and thus are aware of the programme can be a bias to our study as the other employers may have a different perspective.

## Conclusion

The need for professional development and career progression for midwives has been identified in contemporary literature that also identified the need for midwifery representation in executive levels of healthcare. Our study aimed to assess the influence of the Master in SRH on midwives’ career progression. The master’s degree is a capacity building programme that has produced midwives and nurses specialised in sexual and reproductive health. Graduates developed skills and acquired advanced knowledge in research, policy, and management, as well as a postgraduate degree. However, while the programme was also intended to equip graduates with advanced clinical skills, this was not achieved. The Master is considered relevant for the career progression of midwives. Nevertheless, the integration into the health system has proved difficult for some graduates, and roles in the health system remain uncertain due to the unclear graduate profile definition and the subsequent non-recognition and unfamiliarity of employers with the graduates’ skills. By better aligning the master’s degree with labour market demands, health system needs and population health priorities, there is potential to increase the recognition of SRH graduates’ skills and thus potentially increase the attractiveness of the training and more positively impact on graduates’ careers.

## Data Availability

All data generated or analysed during this study are included in this published article.

## Abbreviations

CSREF: Centre de Santé de Référence
DGS: Direction Générale de la Santé
DRS: Direction Régionale de la Santé
ECOWAS: Economic Community of West African States
ENT: Ear, Nose, and Throat
FP: Family Planning
INFSS: Institut National de Formation en Sciences de la Santé
LMD: Licence Master – Doctorate
LMIC: Low-and-Middle-Income Countries
MMR: Maternal Mortality Ratio
MPH: Master of Public Health
NGO: Non-Governmental Organization
RH: Reproductive Health
SDG: Sustainable Development Goals
SRH: Sexual and Reproductive Health
SSI: Semi Structured Interview
WAHO: West African Health Organization
WHO: World Health Organisation
